# Posttraumatic Stress in Parents of Children Diagnosed with Cancer: Hyperarousal and Avoidance as Mediators of the Relationship between Re-Experiencing and Dysphoria

**DOI:** 10.1371/journal.pone.0155585

**Published:** 2016-05-17

**Authors:** Emma Hovén, Lisa Ljungman, Marike Boger, Brjánn Ljótsson, Nicola Silberleitner, Louise von Essen, Martin Cernvall

**Affiliations:** 1 Clinical Psychology in Healthcare, Department of Public Health and Caring Sciences, Uppsala University, Uppsala, Sweden; 2 Department of Clinical Neuroscience, Division of Psychology, Karolinska Institutet, Stockholm, Sweden; Central Institute of Mental Health, GERMANY

## Abstract

**Background:**

Increased understanding of the relationships between different symptom clusters involved in posttraumatic stress symptoms (PTSS) could guide empirical research and clinical practice. The objective of the present study was to investigate whether hyperarousal and avoidance mediated the relationship between re-experiencing and dysphoria in parents of children diagnosed with cancer.

**Methods:**

Longitudinal data from parents of children receiving cancer therapy were used. PTSS were assessed using the PTSD Checklist Civilian Version at one week (T1), two (T2) and four months (T3) after diagnosis. Mediation analyses for multiple mediators were conducted for mothers (*n* = 122) and fathers (*n* = 121), respectively. The mediation model tested the assumption that the PTSS symptom clusters hyperarousal and avoidance mediated the relationship between re-experiencing and dysphoria.

**Results:**

For fathers, none of the hypothesized mediators were significant. For mothers, hyperarousal mediated the relationship between re-experiencing and dysphoria, but avoidance did not.

**Conclusions:**

Results suggest that hyperarousal is important for the development of dysphoria in mothers, supporting use of interventions targeting such symptoms in the early and ongoing period following the child’s diagnosis.

## Introduction

The experience of having a child diagnosed with cancer can be overwhelming and can cause extensive distress for parents [1, 2]. Posttraumatic stress symptoms (PTSS) have been identified as one of the most evident and important psychological consequences for parents of children diagnosed with cancer [3]. PTSS are associated with psychiatric comorbidity [4], neurocognitive deficits [5], and may hamper parents’ capability to make treatment decisions and to provide adequate emotional support to their children [6]. Across studies, clinically significant levels of PTSS have been reported by 22–68% of parents of children on treatment and 11–44% of parents of children off treatment [3, 6–9]. PTSS consist of different types of symptoms such as re-experiencing aspects of the trauma, avoidance of reminders, and emotional and physical symptoms [10]. Despite the number of studies reporting on PTSS in parents of children diagnosed with cancer, there is a lack of research examining the relationships between these different types of symptoms. Such research could increase the understanding of the development of PTSS in parents of children diagnosed with cancer and could guide further empirical research and clinical practice with this population.

In order to increase the understanding of the relationships between different symptoms of posttraumatic stress disorder (PTSD) in this population, one needs to consider how PTSS is best defined in parents of children diagnosed with cancer. In the Diagnostic and Statistical Manual of Mental disorders, 4th edition (DSM-IV), PTSS pertain to three factors or symptom clusters: re-experiencing, avoidance and hyperarousal [11]. However, research based on DSM-IV measurement of PTSS/PTSD supported the construct validity of a four-factor solution of PTSS including the factors re-experiencing, avoidance, hyperarousal, and dysphoria/general distress [12] for a variety of populations [13–16], including parents of children diagnosed with cancer [17]. The four-factor solution includes a broad factor termed dysphoria or general distress, which comprises symptoms similar to the nonspecific symptoms of many depressive and anxiety disorders i.e., emotional numbing, irritability, difficulty sleeping, and difficulty concentrating [12]. Reflecting this emerging evidence, PTSS or PTSD as described in the DSM-5 [10] consists of four factors (intrusions, avoidance, negative alterations in cognitions and mood, and marked alterations in arousal and reactivity). Increased knowledge about the relationships between the four symptom clusters involved in PTSS could elucidate which symptoms to target in interventions aiming to prevent and/or mitigate parental distress during the ongoing and evolving phase of the reaction to a child’s cancer diagnosis.

Two of the core symptoms of PTSS, avoidance and hyperarousal, have been put forth in the literature regarding their potential importance as pathogenic mechanisms of distress. Avoidance of external and internal aversive stimuli has been proposed as central for the development and maintenance of psychiatric symptoms in general [18], and of PTSS specifically [19]. Furthermore, avoidance has been identified as a mediator between re-experiencing and general distress in the Cognitive Processing Model [20]. According to this model, avoidance of reminders of the trauma may increase the level of distress as thoughts and memories are not confronted directly, and thus not efficiently processed. Hyperarousal is a core symptom not only in PTSS, but also in anxiety disorders such as panic anxiety disorder and generalized anxiety disorder [21, 22], and has been shown to mediate the relationship between trauma exposure and physical ill-health symptoms [23–25]. Hyperarousal is defined as a state associated with an exaggerated startle response and symptoms concordant with anxiety, such as increased heart rate and rapid and constricted breathing [26]. Conceptual models regarding the symptom progression of PTSS in different trauma populations suggest that hyperarousal predicts emotional numbing, which is part of the dysphoria factor [27–29], and subsequent PTSD symptom severity [30]. Different explanations have been offered for why hyperarousal has a central role in development and maintenance of psychological distress. It has been argued that the body mobilizes cognitive, behavioral, and emotional resources in order to deal with hyperarousal and if the hyperarousal state is prolonged individuals deplete these resources leading to exhaustion, distress, and emotional numbing [27, 28, 31]. To our knowledge, no study has examined the effect of hyperarousal in parents of children diagnosed with cancer, even though the situation in this population is conceived of ongoing stressors [7] making a prolonged state of hyperarousal plausible.

The purpose of this study was to investigate hypothesized relationships between PTSS symptom clusters in parents of children diagnosed with cancer using longitudinal data in mediation analyses. The hypothesized direct and indirect effects were based on the empirically validated four-factor model of PTSS [12, 17]. Based on the literature reviewed above we hypothesized that hyperarousal and avoidance would mediate the relationship between re-experiencing and dysphoria, i.e., that re-experiencing predicts later dysphoria via the mediating factors hyperarousal and avoidance. This was tested with data collected in a study using a prospective cohort design. Previous research suggests that the mechanisms involved in PTSS might differ between women and men [32, 33], and that mothers of children diagnosed with cancer report a higher level of PTSS than fathers [7, 34]. The analyses were therefore conducted for mothers and fathers separately.

## Materials and Methods

This study is part of a larger project investigating psychological and health economic consequences of parenting a child diagnosed with cancer. The project’s longitudinal design covered seven assessments (T1-T7) from one week after the child’s diagnosis up to five years after the end of the child’s treatment or the child’s death. The present study was based on data collected at the first three assessments: One week (T1), two (T2) and four months (T3) after the child’s diagnosis. T1-T3 were administered to capture parents’ experiences during their child’s treatment. Specifically, T1 was set to capture the experience of receiving the child’s diagnosis; and T2-T3 to capture experiences during the period of active cancer treatment. Data were on average collected the following number of days after diagnosis: 8 (*SD* = 2.2) (T1), 61 (*SD* = 5.9) (T2), and 119 (*SD* = 12.7) (T3).

### Sample

Parents of children treated at four of the six Swedish pediatric oncology centers (Gothenburg, Linköping, Umeå, and Uppsala) were consecutively recruited across 18 months during 2002 through 2004. Eligibility included the following criteria: Swedish- and/or English-speaking parents (including step-parents) of children aged 0–18 years and diagnosed (≤14 days previously) with a primary cancer diagnosis, and scheduled for chemotherapy and/or radiotherapy. Additionally, parents should have contact with the child, be considered by the responsible pediatric oncologist to be physically and emotionally capable of participating, and have access to a telephone. Eligibility also required that that the child was on curative treatment at T2 and T3. The study population consisted of 388 parents of 188 children. Out of the 325 parents who were found eligible for the study, 259 consented to participate at T1, representing an 80% response rate. The most common reason provided by parents declining participation was not being able to prioritize participation under circumstances (*n* = 44). No significant difference was found between participants (*n* = 259) and non-participants (*n* = 63 excluded; *n* = 66 declined) regarding parent or child age. However, more parents of a child with a CNS tumor (52%) were excluded/declined participation (χ^2^ = 14.60, *p* = 0.001). The majority of these were excluded because our research group was not able to approach them within 14 days after diagnosis. Out of the 259 participating parents, 243 parents provided data at T2 (*M* = 61.2 days after diagnosis, *SD* = 4.3), and 214 parents provided data at T3 (*M* = 120.2 days after diagnosis, *SD* = 5.0). As the current mediation analysis requires data from at least the first two assessments, the 16 participants who only provided data at T1 were excluded. The excluded participants did not significantly differ from the remaining 243 participants regarding level of PTSS at T1, age, and gender (parent and child), marital status and type of cancer. Descriptive statistics for the 243 parents (122 mothers/121 fathers), representing 132 families, are presented in Table 1. At T3, 214 parents (107 mothers/107 fathers) participated. The most common reason for attrition at T3 was that the child had ended successful treatment (*n* = 24). Other reasons for exclusion at T3 were palliative care/death of the child (*n* = 2) and that the child moved to a non-participating pediatric oncology center (*n* = 1). Only 2 parents declined participation at T3 (1 not interested/1 too emotionally moved by the last interview).

**Table 1:** Parent and Child Characteristics.

Characteristic	Mothers		Fathers		Children	
	(*n* = 122)		(*n* = 121)		(*n = 132)*	
	n	%	n	%	n	%
**Parent of daughter/son**	55/67	45.1/54.9	55/66	54.5/45.5		
**Marital status**						
Spouses/couples	112	91.8	113	93.4		
Single	10	8.2	8	6.6		
**Education**						
Basic (≤9 years)	11	9.0	23	19.0		
Secondary	61	50.0	66	54.5		
Post secondary (>14 years)	48	39.3	30	24.8		
Not stated	2	1.6	2	1.7		
**Age, years**						
< 30	19	15.6	10	8.3		
30–39	63	51.6	62	51.2		
≥ 40	40	32.8	49	40.5		
**No. of siblings**						
0					13	9.8
1–2					97	73.5
≥ 3					22	16.7
**Age, years**						
0–3					37	28.0
4–7					35	26.5
8–12					34	25.8
13–18					26	19.7
**Type of cancer**						
Leukemia/lymphoma					52	39.4
CNS tumor					16	12.1
Other solid tumor					64	48.5

### Measures

#### Background information

Demographic information pertaining to e.g., civil status, parent age and number of siblings of the child diagnosed with cancer, were collected at each assessment. Medical data and parent educational level were recorded at T1.

### Posttraumatic stress symptoms

PTSS were assessed with the Swedish version of the PTSD Checklist Civilian Version (PCL-C) containing 17 items that map directly onto the DSM-IV PTSD symptom clusters [11, 35]. The respondents were asked to rate the extent to which they had been bothered by each symptom during the previous week (at the T1 assessment) or month (at the T2-T3 assessments) on a 5-point Likert scale ranging from “not at all” (1) to “extremely” (5). Items were keyed to the child’s cancer disease. The total score ranges from 17 to 85, with a total score of >44 indicating PTSS corresponding to a clinical PTSD diagnosis [36]. The PCL-C measure has demonstrated robust psychometric properties with adequate internal consistency for the full scale, test-retest reliability, and evidence for good convergent and discriminant validity when compared to other well-established PTSS measures [37].

The analyses were conducted based on the four-factor model of PTSS [12, 17] entailing intrusion/re-experiencing (items 1–5), avoidance (items 6, 7), hyperarousal (items 16, 17), and dysphoria/general distress (items 8–15). Since a recent report [17] of the study population denoted acceptable internal consistency of the four-factor model with the exception of the avoidance factor, which evidenced poor internal consistency, the two items of avoidance were used as separate entities in the mediation analyses. This allowed for an examination of the potentially different roles of external (item 6) and internal (item 7) avoidance.

The alpha coefficients of the PTSS factors at T1 to T3 ranged from 0.68–0.82 (re-experiencing), 0.70–0.79 (hyperarousal), and 0.75–0.83 (dysphoria).

### Procedure

Parents who met the inclusion criteria were provided written and oral information about the study by a coordinating nurse at the respective center within the first two weeks after the child’s diagnosis. The same nurse asked parents for oral informed consent to participate and permission to be contacted over the telephone by a research assistant. The research assistant conducted the interview via telephone where demographic data, the PCL-C and other instruments (not reported herein) were administered. At the end of each interview (T1-T2) oral consent to contact the parent again (T2-T3) was acquired by a research assistant. Oral consent was obtained in accordance with the standards for informed consent for data collection via telephone at the time the study was conducted. Consent was documented by the coordinating nurse at T1 and thereafter by the research assistant who conducted the respective interview. Ethical approval of the study procedure, including the process to obtain consent, was obtained in 2002 from the local research ethics committees at the respective faculties of medicine in Gothenburg (Research Ethics Committee, Faculty of Medicine, University of Gothenburg), Linköping (Research Ethics Committee, Faculty of Medicine, Linköping University), Umeå (Research Ethics Committee, Faculty of Medicine and Odontology, Umeå University), and Uppsala (Research Ethics Committee, Faculty of Medicine, Uppsala University) when the study was launched (DNR:02–006). In 2004 the organization of ethical vetting in Sweden changed, from being administered by local ethical research committees to being administered by regional ethical committees. The study procedure including the procedure to obtain consent was approved by the Regional Ethical Review Board in Uppsala in 2008 (DNR: 2008/109).

### Statistical analyses

**Fig 1:**
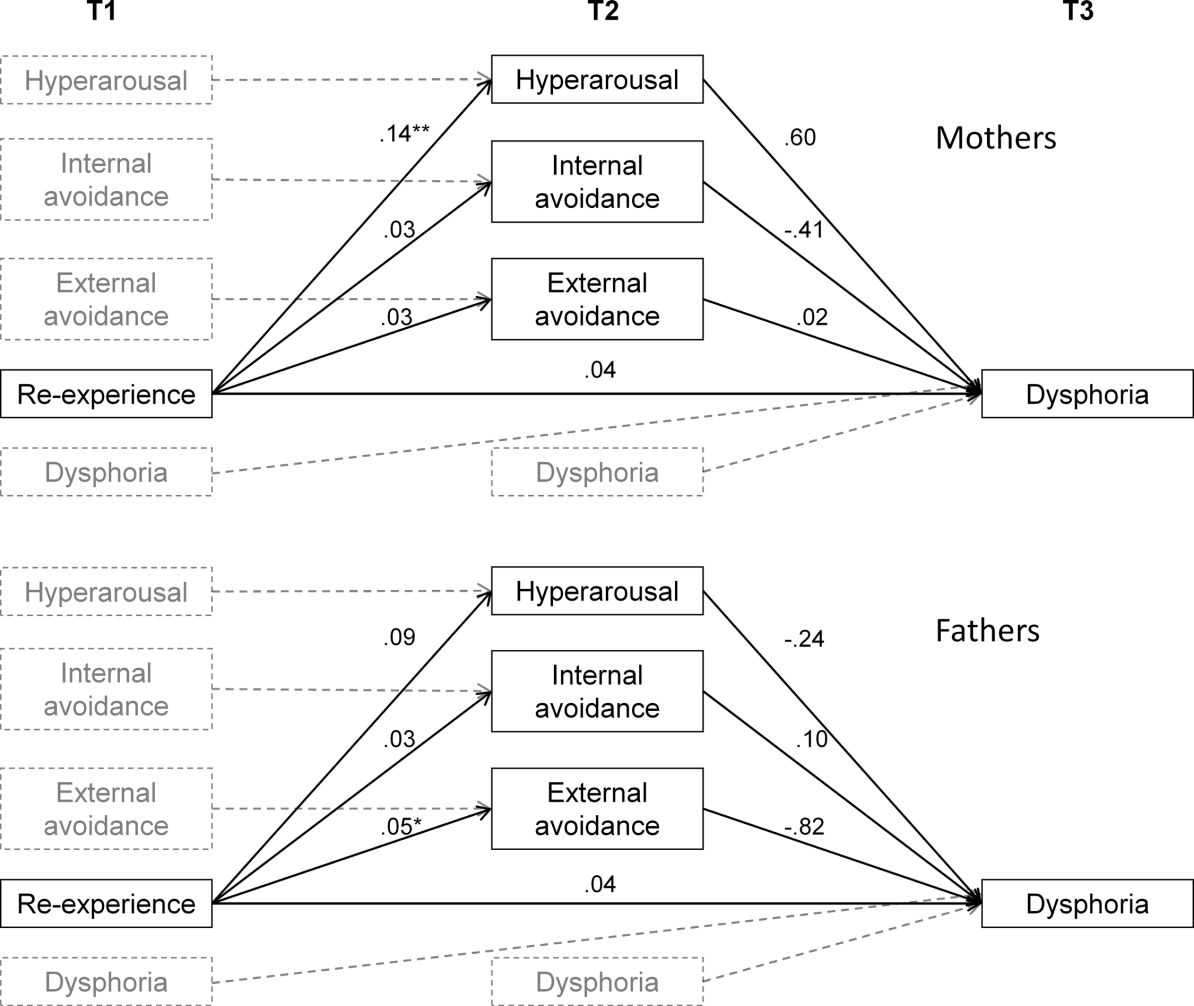
Estimates of direct effects for mothers and fathers respectively. The included covariates are marked with dashed lines. * *p* <0 .05 ** *p* < 0.01.

Descriptive statistics were calculated using SPSS Statistics Version 22.0, Chicago: SPSS Inc. Differences between mothers’ and fathers’ reports of re-experiencing internal/external avoidance, and hyperarousal were assessed by *t*-tests. To examine the effect of the independent variable on the dependent variable via the mediating variables, mediation analyses with multiple mediators were performed with observed variables using Mplus 6.1 [38]. The analyses were performed using maximum likelihood estimation of direct and indirect effects. Bootstraps (*n* = 5000) with 95% confidence intervals (CI) were used to evaluate the significance of the indirect effects. In the analyses, re-experiencing at T1 was included as the independent variable; external avoidance, internal avoidance, and hyperarousal at T2 were included as mediators; and dysphoria at T3 was included as dependent variable. Previous values of the mediators and the dependent variable were included as covariates, see Fig 1. Analyses were performed for mothers and fathers separately. Using maximum likelihood estimation, missing data at T3 was assumed to be missing at random.

## Results

Descriptive statistics for the separate factors of PTSS at each assessment are presented in Table 2, and correlations between the main variables included in the mediation analyses are presented in Table 3. As is evident from Table 3, there were significant correlations between all of the main variables included in the mediation analyses.

**Table 2:** Mothers’ and Fathers’ Reports of PTSS at T1-T3.

	T1			T2			T3		
Factors	Mothers	Fathers		Mothers	Fathers		Mothers	Fathers	
	(*n* = 122)	(*n* = 121)		(*n* = 122)	(*n* = 121)		(*n* = 107)	(*n* = 107)	
	*M* (*SD*)	*M* (*SD*)	*p* value	*M* (*SD*)	*M* (*SD*)	*p* value	*M* (*SD*)	*M* (*SD*)	*p* value
Re-experiencing	2.47 (0.91)	2.05 (0.68)	<0.001	2.32 (0.89)	1.87 (0.69)	<0.001	2.19 (0.92)	1.62 (0.58)	<0.001
Hyperarousal	2.81 (1.21)	2.38 (1.02)	0.003	2.58 (1.12)	1.91 (0.92)	<0.001	2.29 (1.08)	1.85 (0.75)	<0.001
Internal avoidance	2.08 (1.27)	1.78 (1.05)	0.043	1.96 (1.20)	1.65 (1.01)	0.032	1.97 (1.16)	1.59 (0.94)	0.009
External avoidance	1.79 (1.24)	1.56 (0.94)	0.120	1.57 (1.00)	1.36 (0.99)	0.046	1.72 (1.07)	1.29 (0.67)	<0.001
Dysphoria	2.56 (0.81)	2.11 (0.60)	<0.001	2.42 (0.79)	2.10 (0.72)	<0.001	2.24 (0.80)	1.94 (0.64)	0.003

*P* value for differences between mothers and fathers as determined by *t*-tests.

**Table 3:** Spearman Correlations between the Main Variables Included in the Mediation Analyses.

	T1			T2								
	Re-experiencing			Hyperarousal			Internal avoidance			External avoidance		
	Total	Mothers	Fathers	Total	Mothers	Fathers	Total	Mothers	Fathers	Total	Mothers	Fathers
**T2**												
Hyperarousal (*n*)	0.52*** (243)	0.56*** (122)	0.44*** (121)									
Internal avoidance (*n*)	0.21*** (243)	0.20* (122)	0.18* (121)	0.30*** (243)	0.27** (122)	0.29** (121)						
External avoidance (*n*)	0.33*** (243)	0.31** (122)	0.38*** (121)	0.40*** (243)	0.42*** (122)	0.37*** (121)	0.31*** (243)	0.30*** (122)	0.30*** (121)			
**T3**												
Dysphoria (*n*)	0.46*** (214)	0.48*** (107)	0.40*** (107)	0.59*** (214)	0.67*** (107)	0.48*** (107)	0.22** (214)	0.20* (107)	0.19* (107)	0.27*** (214)	0.30** (107)	0.22* (107)

* *p* < 0.05

** *p* < 0.01

*** *p* < 0.001.

Results of the direct effects from the main mediation analyses for mothers and fathers are presented in Fig 1. The indirect effects of the hypothesized mediators are presented in Table 4.

**Table 4:** Estimates of the Indirect Effects in the Mediation Analyses on Dysphoria.

Mediator	Estimate	95% CI
**Mothers**		
Hyperarousal	0.084	0.011–0.222
Internal avoidance	-0.011	-0.058–0.004
External avoidance	0.001	-0.043–0.054
**Fathers**		
Hyperarousal	-0.022	-0.100–0.007
Internal avoidance	0.003	-0.016–0.043
External avoidance	-0.042	-0.142–0.011

CI = confidence interval.

For mothers, there was a direct effect from re-experiencing at T1 to hyperarousal at T2 (Fig 1). No other significant direct effects were found while controlling for initial levels of the main variables. The tests of the indirect effects, i.e., mediation, revealed that none of the avoidance factors at T2 mediated the relationship between re-experiencing at T1 and dysphoria at T3 (Table 4). However, hyperarousal at T2 was a significant mediator of this relationship (Table 4). The proportion of explained variance in dysphoria at T3 was 58% for the full model including all covariates.

For fathers, there was a significant direct effect from re-experiencing at T1 to external avoidance at T2 but no other significant direct effects while controlling for initial levels (Fig 1). None of the hypothesized mediators were significant (Table 4). The proportion of explained variance in dysphoria at T3 was 67% for the full model including all covariates.

## Discussion

The main goal of this study was to prospectively examine the relationships among symptom factors of PTSS in parents of children on cancer treatment. The specific aim was to investigate if avoidance and/or hyperarousal mediate/s the relationship between re-experiencing and dysphoria in mothers and fathers, respectively. The results did not support the hypothesis with regard to avoidance as a mediator in the relationship between re-experiencing and dysphoria, neither for mothers nor fathers. However, results supported the hypothesis of hyperarousal as a mediator in the relationship between re-experiencing and dysphoria for mothers, but not for fathers.

Among fathers, re-experiencing predicted subsequent external avoidance but external avoidance did not predict subsequent dysphoria. Despite the direct association of re-experiencing on external avoidance the magnitude of this effect appears modest viewing that external avoidance did not mediate the relationship between re-experiencing at the time of diagnosis and later dysphoria. The null finding of the mediating role of internal and external avoidance among mothers and father corresponds to previous results with populations exposed to recent and ongoing medical trauma [39]. Although assessed with single-items, our findings indicate that avoidance, even though well studied as to its pathogenic effects in other trauma populations [19, 20], does not necessarily have a maladaptive function for parents during the period shortly after a child’s cancer diagnosis. These results corresponds to previous findings [20, 39, 40] indicating that early in a child’s illness trajectory, avoidance, as long as it is not performed excessively, could even be helpful in regulating the amount of information and emotional reactions processed, while it might become less adaptive in the long term. It should be noted that the direct effect of re-experiencing on external avoidance was significant among fathers and this might indicate that fathers are more prone to external avoidance in response to intrusive thoughts and memories than mothers. However, the current results do not support a mechanism where such avoidance leads to dysphoria. Future studies are warranted to more clearly determine if the effects of avoidance in mothers and fathers of children with cancer are dependent on time since diagnosis and whether there are gender differences in this potential dependency.

Hyperarousal was found to mediate the relationship between re-experiencing and dysphoria in mothers. The mediating role of hyperarousal is in accordance with findings for other trauma populations [27–30]. However, hyperarousal did not mediate the relationship between re-experiencing and dysphoria in fathers. Several reasons could account for the seemingly contradictory role of hyperarousal in mothers and fathers. A gender difference in the stress response is one possible explanation. This has been put forth in earlier research where women in motor vehicle accidents were found to be at greater risk than men for intense feelings of distress and physical reactivity even though the trauma itself and the amount of re-experiencing was equivalent [32]. The authors argued that this might be the result of a greater sensitivity to contextual and memory-linked arousal in women, which could sustain arousal states. Differences in stress response are also evident in mothers and fathers of children diagnosed with cancer, with a higher level of PTSS reported by mothers than fathers from shortly after diagnosis up to five years after end of treatment [7, 34]. Another explanation relates to differences in the gender role related strains experienced by mothers and fathers of children with severe health conditions. For example, mothers of children with cancer more often stay at the hospital and support their child through painful treatments and adverse side effects. Also, mothers of children diagnosed with cancer have been found to be on sick leave to a larger extent than fathers, both during and after the end of treatment [41]. There is however research showing that fathers report the same levels of distress as mothers when they are the primary caregiver, supporting the gender role explanation of the differences found between mothers and fathers [7, 42, 43]. Although not directly assessed, the results in the present study may imply that mothers were the primary caregivers to a greater extent, and the mediating effect of hyperarousal might thus reflect differences in caregiving patterns rather than a gender difference per se.

Some limitations of the present study need to be addressed. Internal and external avoidance was measured with just one item each, which might have influenced the reliability of the avoidance indicators. Future studies seeking to examine the potential mediating role of avoidance should preferably assess avoidance in a broader manner with multiple questions targeting parents’ use of internal and external avoidance strategies. Furthermore, to achieve greater power in the analyses it would have been beneficial to include both mothers and fathers in one main analysis with gender included as a potential moderator of the indirect effects. However, due to the dependency of mother and father dyads and previous research indicating that the mechanisms involved in PTSS might differ between women and men [32, 33] we conducted separate analyses for mothers and fathers.

## Conclusions

This study provides a more nuanced understanding of the development of PTSS in parents of children on cancer treatment by investigating whether the relationship between re-experiencing and dysphoria is mediated by hyperarousal and avoidance in parents of children undergoing active cancer treatment. The results suggest that avoidance, as measured in the present study, is not a significant mediator in the relationship between re-experiencing and dysphoria. However, hyperarousal is suggested as a significant mediator through which re-experiencing leads to dysphoria among mothers, but not among fathers. Reducing hyperarousal symptoms in the early and ongoing period following a child’s diagnosis holds potential promise of influencing the course of development of dysphoria/general distress in mothers. Hyperarousal can be targeted by e.g., relaxation training, stress management and/or mindfulness-based exercises and interventions including such components could be a viable option to reduce mothers’ distress in response to their child’s cancer. A recent report suggests that guided self-help via the Internet including relaxation training shows promise in reducing PTSS and depression in parents of children on cancer treatment [44]. However, additional empirical evidence to support the efficacy of such interventions in the current population is needed. Further research of the development and maintenance of PTSD in mothers and fathers is needed to determine possible gender differences, taking into account family characteristics and family caregiver roles. Finally, to replicate and corroborate the present findings, future studies are warranted on the relationship among symptom factors of PTSS in parents of children diagnosed with cancer.
